# Impact of the COVID-19 Health Crisis on Trans Women and Cis Men Sex Workers in Spain

**DOI:** 10.1007/s10508-022-02405-5

**Published:** 2022-09-06

**Authors:** Juan M. Leyva-Moral, Juliana Castro Ávila, Marta Villar, Beti López, Héctor Adell, Mercè Meroño, Kevin Santander, Laia Ferrer, Jocelyn Mesías-Gazmuri, Rocío Astudillo Alonso, Daniela Rojas Castro, Jordi Casabona, Cinta Folch

**Affiliations:** 1 Nursing Department, Faculty of Medicine, Grup de Recerca Infermera en Vulnerabilitat i Salut (GRIVIS), Universitat Autònoma de Barcelona, 08193 Bellaterra, Spain; 2 Coalition PLUS, Paris, France; 3 Stop Sida, Barcelona, Spain; 4 Fundació Àmbit Prevenció, Barcelona, Spain; 5 NGO Stop Sida, Barcelona, Spain; 6 Clinical Instructor at Nursing Department, Universtiat Autònoma de Barcelona, Barcelona, Spain; 7 Departament de Salut, Generalitat de Catalunya, Centre d’Estudis Epidemiològics Sobre Les Infeccions de Transmissió Sexual i Sida de Catalunya (CEEISCAT), Badalona, Spain; 8 Departament d’Igualtat i Diversitat Ciutadana, Ajuntament de Mataró, Mataró, Spain; 9 Institut d’Investigació Germans Trias i Pujol, Badalona, Spain; 10 Departament de Salut | Generalitat de Catalunya, ASSIR Esquerra Barcelona, Servei Trànsit Barcelona. Institut Català de La Salut, Barcelona, Spain; 11 Coalition PLUS, Paris, France; 12 Centro de Investigación Biomédica en Red de Epidemiología y Salud Pública (CIBERESP), Madrid, Spain

**Keywords:** Sex work, COVID-19, Determinants of health, Transgender, Trans women, Qualitative research

## Abstract

The objective of the study was to describe the impact of the COVID-19 pandemic on sex workers in accessing health and social services. A qualitative study was conducted using semi-structured interviews with 29 participants in Barcelona, Spain. Data were analyzed using thematic analysis. Four themes were identified: (1) impact of COVID-19 on physical/mental health, (2) barriers and facilitators to health/social service access, (3) health decision-making, and (4) suggestions for future pandemic situations. Barriers to accessing health services were structural. Non-governmental organization support was the main facilitating factor. A person-centered, intersectional approach is suggested for future practice, considering co-occurring syndemic factors.

## Introduction

Trans women (TW) and cisgender men (CM) sex workers (SW) have a high burden of human immunodeficiency virus (HIV) and other sexually transmitted infections (STIs) worldwide. It is higher than that of other SW, suggesting the existence of unique challenges for this group when accessing healthcare (Beyrer et al., 2015). Specific barriers to accessing the health system include stigma and discrimination, in addition to the scarce education that some health professionals have concerning working with such communities (Brookfield et al., 2020; Leyva-Moral et al., 2020, 2021). HIV and other STI screening, counseling, and treatments that are accessible in health services are poorly adapted to the needs of SW (Brookfield et al., 2020). Consequently, these people experience different types of discrimination due to their sexual and gender identity or the stigma related to sex work (Baral et al., 2015; Brookfield et al., 2020). Furthermore, HIV-related stigma is an additional barrier to HIV testing, leading to delayed diagnosis and access to treatment (Brookfield et al., 2020). On the other hand, the limited civil, political, economic, social, and cultural rights that migrants have, guarantees the marginalization of migrant SW, as their labor is unrecognized, if not directly criminalized, in host countries (Global Network of Sex Work Project, 2018). In this sense, their immigration and social status as part of an ethnic minority must be considered in addition to the different forms of discrimination they experience (Earnshaw et al., 2013; Logie et al., 2011). The European Network for HIV/STI Prevention and Health Promotion among Migrant Sex Workers emphasized that migrant SW have greater difficulty accessing health and social care due to social exclusion or isolation (TAMPEP, 2019).

Members of key populations, including SW and transgender people, were disproportionally impacted by COVID-19 (Meeting Targets and Maintaining Epidemic Control (EpiC), 2020). As one of the most marginalized communities, SW are being left behind, as the measures adopted by the governments of Spain and Catalonia to face the health emergency caused by COVID-19 (lockdown and social distancing) did not consider the needs of these populations. This can also be seen in many other countries (Jozaghi & Bird, 2020; Platt et al., 2020; UNAIDS, 2020b). Lockdowns, self-isolation, and travel restrictions prevented them from working. Some were even forced onto the streets and into destitution, where the risks were heightened by the pandemic (ICRSE, 2020). For this reason, they had to resort to charity and aid provided by NGOs to cover some of their essential needs (Bernabé, 2020). In this context, new barriers to health services access have appeared, such as the closure of sexual health services or interruptions in the distribution of condoms or other sex practice-related materials, among others (Álvarez García, 2020). Few studies have examined the social and structural determinants that affect access to the health system by TW and CM SW. Therefore, the impact COVID-19 may have on access to prevention and treatment services remains unknown (Beyrer et al., 2015; Shannon et al., 2015). The analysis of barriers and facilitators to health service access for TW and CM SW requires approaches that go beyond individual factors, making it necessary to cover a greater diversity of experiences and needs through a research methodology that captures this complexity (Levesque et al., 2013; Ma et al., 2017; Wanyenze et al., 2017). Qualitative methodologies have previously been used successfully to identify and describe barriers to accessing social and health services (Sevelius et al., 2016). It must be noted that sex work in Spain is legal, however not recognized as a job from which access to free health service, sickness leave and other rights recognized for all workers. This study aims to describe the perceived impact of the COVID-19 pandemic on TW and CM SW, as well as to assess the barriers and facilitators to health and social service access during the pandemic.

## Materials and Methods

This qualitative exploratory study was approved by the Germans Trias i Pujol University Hospital Ethics Committe (Badalona, Spain). This was conducted between November 2020 and February 2021 using the framework of the SexCohort project, a longitudinal study of TW and CM SW. This study was performed in collaboration with the EpiC Project, a multi-country, cross-sectional, community-based study assessing the impact of the COVID-19 health crisis on populations at risk to or living with HIV and hepatitis C virus (HCV), and on people who work with these populations in community settings. TW and CM SW aged 18 years or older were purposively recruited from two community-based centers in Barcelona. NGOs workers informed clients about the study and provided information about how to participate. The qualitative methodology allowed us to approach the phenomenon of the study in its entirety and helped us understand how it manifests itself and what factors are related (Polit & Beck, 2017). Data were collected in Spanish through semi-structured face-to-face interviews. Online interviews were also given as an option due to the COVID-19 pandemic; however, the participants were not amenable to this option. The interview script adapted from the EpiC Project was created by consensus among the members of the research team and the community leaders. Several discussion sessions were required until the final version was completed. Given the methodology used, the script was open to modifications and adaptations (Polit & Beck, 2017). Open-ended questions explored the following: (a) knowledge and perception of COVID-19, (b) experiences of sex work during lockdown, (c) specific health and social needs, (d) knowledge of available help resources, (e) access to comprehensive medical care, (f) resources search strategies, (g) main perceived barriers to access to care services, (h) sex work experiences, (i) discrimination in social or health services, and (j) experiences of institutional and individual violence. The sample questions are provided in Table 1.

**Table 1:** Sample questions from the semi-structured interviews

1. How would you describe your current physical and emotional health?
2. What worries you about your current state of health? Why? Have your health concerns changed due to the COVID-19 health crisis? How?
3. In what way do you think COVID-19 has changed the way you use health services?
4. What have you missed during this time of pandemic and lockdown related to your health and social needs?
5. How was the care received during the pandemic? Have you accessed telemedicine consultations? How was it?
6. Tell me about your day-to-day time since the beginning of the crisis and/or confinement. How did you feel during confinement?
7. How do you think COVID-19 has affected sex work? Why?
8. What impact does this health crisis have on discrimination and stigmatization toward sex workers?
9. What do you think of the measures adopted by the Government to face the COVID-19 health crisis?
10. What could NGOs do to better adapt to the needs of sex workers during the COVID-19 health crisis?

The interviews were conducted at the facilities of two communities in Barcelona—(hidden for blinding purposes)—by three health technicians trained for this purpose by the principal investigator (PI). The interviews were audio-recorded and transcribed verbatim immediately afterward. Field notes, which mainly included observations, reflections, and clarifications during the interviews discussed with the PI, were also collected and included in the data analysis. In addition, the PI met with the interviewers periodically to resolve doubts or incidents, verify that the interviews were understood, and the data collected were relevant. The average duration of the interviews was 45 min. Participants were recruited intentionally and received an incentive of 20 euros.

Before starting the interviews, the participants received detailed information about the study and were able to ask questions before signing the informed consent. No identifying data were collected. Moreover, the names of the participants were replaced by an alphanumeric code (Participant 1 = P1 and so on). Interviews were conducted until the data were saturated (i.e., when the data collected and analyzed did not offer new results, informational redundancy occurred) (Sandelowski, 2008). To have a diverse sample, years of practicing SW, the administrative situation in Spain (regular, irregular, or pending), HIV and hepatitis C serological status, and gender identity (cis, trans, queer, etc.) were taken into account.

Data were analyzed thematically following the method proposed by Braun and Clarke (2012) with the support of the Atlas.tiV8 software. Thematic analysis is an approach for analyzing qualitative data to answer broad research questions. It is a flexible analytical method that does not pretend to be neutral since researchers may influence any analysis. Thus, the final product is one or more themes that explain people’s experiences, perceptions, and points of view regarding the phenomenon studied (Braun & Clarke, 2012). Verbatims were translated into English and verified by two bilingual research team members to insure the translated verbatims retained not only syntax but also the original meaning. First, several readings were made to become familiar with the content of the data. Second, descriptive codes were assigned to the different identified contents. Third, these codes were grouped into categories based on their similarities and differences. Finally, the results obtained were discussed by the research team in several discussion sessions until an agreement was achieved. The Consolidated Criteria for Reporting Qualitative Research (COREQ) checklist was used in this study (Tong et al., 2007).

Throughout the process, the rigor criteria proposed by Guba (1981) and Guba and Lincoln (1994) were considered. The entire research process was closely and continuously monitored to achieve reliability, paying attention to the accuracy of the transcripts and comparing them with their corresponding audios. Similarly, the transcripts were read several times to guarantee the fidelity of their contents. To verify the veracity and realism of the categories identified, three experts in qualitative methodology that were not members of the research team reviewed and verified the analytical process and the results, which had also been previously agreed upon with the experts on the topic. All authors were involved in working with vulnerable populations and some of them are visible members of the LGBTQIA + community. Four researchers were actively caring for sex workers during data collection. Coordination meetings were held to discuss how personal identities and occupations of the research team could shape data collection and analysis. Efforts were made to identify personal assumptions and preconceptions and bracket them. Constant access to the principal investigator was provided to debrief and discuss any doubt or concerns.

## Results

Twenty-nine CM and TW SW were interviewed. Their main socio-demographic characteristics are shown in Table 2. Four themes were identified in the data regarding the impact of COVID-19 on their health status and access to health and social care. Exemplar quotations were chosen to illustrate these themes.

**Table 2:** Participants’ characteristics

Age^a^	33.9 ± 8.4	
Years as a sex worker^1^	9.9 ± 9.4	

	*n*	%
Gender		
Trans woman	20	69.0%
Cis man	6	20.7%
Queer	2	6.9%
Non-binary	1	3.4%
Country of origin		
Latin America	26	89.7%
Spain	3	10.3%
Arrival to Spain		
After 2010	5	19.2%
Before 2010	21	80.8%
Immigration status		
Regular	12	41.4%
On-going	11	37.9%
Non-regular	6	20.7%
Health insurance		
Government	26	89.7%
Private + Government	3	10.3%
HIV status^b^		
Positive	19	65.5%
Negative	10	34.5%
HCV status VHC^c^		
Negative	27	93.1%
Unknown	2	6.9%

^a^Mean ± Standard Deviation; ^b^Human Immunodeficiency Virus; ^c^Hepatitis C Virus

### Physical and Mental Health Challenges Faced During COVID-19

The participants stated that the confinement and pandemic harmed their emotional health. Their socialization and adaptation patterns have changed, and loneliness has been a part of the new situation in many cases. Labor and economic difficulties have also generated psychological complications. Emotional fragility is evident given the uncertain future concerning their employment situation as SW and their economic situation. In addition, they have a difficult situation due to their profession, in which they had to live daily with structural violence due to their multiple risks (such as non-regularized work, job instability, migrant status). This situation has been accentuated during the COVID-19 pandemic, directly impacting the difficulty of working and maintaining oneself.

Emotionally, there are moments when I feel a bit down and melancholic because of the situation we are living in now. It is more on the emotional side. Physically, I see myself, and I consider myself to be doing well. (P11. Trans woman)

I am concerned about the situation. The bars are going to close; they will possibly close everything on Friday, which makes me think more about what we have to do. We will have to go out and find lives ourselves. We do not have any help. (P3. Trans woman)

Both confinement and the pandemic itself have negatively impacted the mental health of TW and CM SW in Barcelona. First, social distancing forced them to separate from their loved ones. Moreover, the possibility of COVID-19 transmission and the fear of infecting other people prompted self-isolation. Episodes of anxiety, depression, mistrust, anguish, sadness, loneliness, boredom, emotional lability, and fear were described. Two of the participants used drugs during this period. Their accounts were completely opposite: one substantially reduced consumption due to the isolation, while the other initiated and maintained it as it was their only way of managing sadness.

When [confinement] began, I felt very depressed. I did not want to get [sick] or have someone else taken from me. But then, you feel bad and experience a low mood, and you’re stuck in the house. The house falls on you. (P9. Trans woman)

During the confinement, I felt very sad and stressed because of my situation and my family’s and friends’ situations. People were dying worldwide, and I was very scared. I was also upset because I couldn’t work and pay my rent. Then I tried drugs [tina], and that’s where it all started. (P5. Cis man)

Naturally, the participants struggled to get out of the negative emotional situation using their personal and professional resources. Their sense of hope helped them continue with life and cope effectively with the situation. They longed to return to their pre-pandemic social interaction, reunite with their friends, enjoy their families, and get back to work and earn an income again. They also communicated with their loved ones through video calls. It made their days more bearable.

I started talking to my friends. We had video calls with several girls, and we talked about things there. It was nice to share our stories when we were disconnected from most of the outside situation. (P1. Trans woman)

The measures implemented by the Government of Spain and Catalonia, mainly lockdown and social distancing, were described as unsatisfactory. The needs of SW were not considered since it is a non-legal profession. Moreover, the closure of bars and nightclubs along with other measures to reduce social interaction directly affected their work and generated a loss or significant reduction in income. These decisions made them feel discriminated against, an experience they have also felt within the neighborhood, specifically when they take clients home or go out to exercise. Some of the participants also employed teleworking; however, it was not widely practiced.

[The government has] not taken us into account, honestly. In other words, there was no noticeable plan to help [during lockdown], nor was it mentioned to us. They never thought about what we’d eat, how we’d pay the rent, or how we’d help our children or our family. They were ready to let the “whore” fall as if she were not a person and didn’t have needs. (P4. Trans woman)

Despite the conditions experienced by SW during confinement and the pandemic, generally, physical deficiencies were not felt, except weight gain and headaches. Some participants even stated that the pandemic became an opportunity to perform better health self-care and adopt healthier lifestyle habits.

I looked for ways to entertain myself within those four walls by exercising, reading, and getting up a little earlier. (P13. Trans woman)

### Challenges and Successes Accessing Health and Social Services

Aside from some exceptional experiences, confinement negatively impacted their access to prevention and health services, particularly regarding medical visits that were canceled or redirected by telephone. In some cases, access to pre-exposure prophylaxis (PrEP) was difficult since the prescribing entities were closed.

It was impossible to get an appointment for the GP – impossible. I had to get a shot for my hormone issues, and they told me that I had to call them. It was really difficult to access them. (P18. Trans woman)

Before COVID-19, I had planned to start taking PrEP, and my first visit was 15 days ago, it took a long time – like four months – for them to get me an appointment. So, my plans for my sexual health were disrupted due to the crisis that we are going through. (P12. Trans woman)

Most participants indicated that COVID-19 had little impact on their physical health. Their follow-up and control visits with family doctors, nurses, and other specialists were substantially reduced or replaced by telemedicine, which was well-received despite their preference for the traditional face-to-face format. The dispensing of medicines was also easier due to telemedicine since treatment prescriptions for antiretroviral, hormone, and antihypertensive drugs could be renewed without incident. Social, emotional, and health incidents were covered by NGOs given that the social services had collapsed and since SW already knew their effectiveness and how to access their services.

I got a good response from the hospital. Visits had been canceled, but they contacted me via email and phone. One time, the pills were missing, but I could go to the pharmacy to pick them up. [NGOs] already do it and are always in a good mood. [They] are there to offer things. And I think the Government should help [them] more for everything, [they] do because [they] do a very good job. [They] are very concerned about people. (P5. Cis man)

The main barriers described by the CM and TW SW were their non-regular immigrant status and waiting lists for health service access. However, these aspects were not exclusive to the pandemic since they were already present before the pandemic. The public health system was described positively; however, access to it was long and complex.

All immigration procedures and appointments stopped [during COVID-19 pandemic]. I could not renew my card or add my fingerprint. I was due in June, and I have been trying to book an appointment these past months through the internet, and nothing happened. Everything stopped. (P14. Cis man)

The support received by the participants from NGOs solved most of their basic needs efficiently (e.g., case management, food, housing, and bureaucracy for changing their name). They also received help from the state social workers, mainly financial and help with health insurance procedures. However, these experiences have not always been satisfactory, mainly due to transphobic situations or the system’s slowness. Despite the difficulties experienced, they appreciated the help extended since they were aware of the seriousness of the problem and the lack of resources and professionals.

The city council also treats me well now, because I had many problems before. The social worker did not treat me as a trans woman; she treated me as a man. I don’t know why – maybe because I had no experience. (P18. Trans woman)

An NGO helped us too. Well, they gave us food and helped us when the coronavirus quarantine was at its peak. It was a sex workers’ union. (P1. Trans woman)

Thank you for always attending to me. You are always there when I write; thanks to you and the NGO, I have all we talked about. I can only thank you since you help those that the Government does not help. You do not have it easy at all, from what I have heard. (P5. Cis man)

Despite the difficulties encountered, satisfaction with the current healthcare system is generally very high. Their appreciation for having a healthcare system that meets their physical, emotional, and social needs, in addition to sexual health, is evident. Satisfaction with health professionals was also high. Except for a specific episode of transphobic discrimination, the public system solved their health doubts and offered them prevention and cure options in most cases. Many of the participants came from countries where access to health services was difficult and slow. Therefore, the system’s failures were minimum compared with the health systems of their countries of origin.

I have had a good experience with health professionals, and they advise me on many things. (P29. Cis man)

It is difficult to make a comparison when you come from a country with so many needs to a country with higher coverage. So, it’s hard to compare, you understand? Because when you come to a country where there are so many things, there is a lot of bureaucracy for everything. If you need an appointment, you must stand in line, because there are many things. So, it seems to me that I see progress. Why? It is not conformism; I simply consider my quality of life to be improved since I have been in Spain. (P7. Trans woman)

### Engagement in Health Risks and Protective Behaviors

Although some CM and TW SW performed some service at home to counteract the economic deficiencies produced by health measures, they clarified that they followed the preventive measures indicated by health authorities. Even among those who received clients at home, measures were employed as far as possible according to the proposed indications, such as hand hygiene, use of hydroalcoholic gel, and ventilation. These preventive measures were continuously carried out even when the confinement period ended since the pandemic is still underway.

I use alcohol. I ask the client to wash himself or take a shower, if necessary. I give clean towels to each client. I also change the sheets. So far, I have not had any symptoms. (P13. Trans woman)

I went back to work in May but, obviously, with the precautions, using alcohol, hand disinfection, and all those kinds of things. As I say, all for biosecurity. (P19. Trans woman)

The isolation measures had a direct negative impact on the participants’ labor sphere. The social distance imposed led to a loss of jobs and a significant economic decline that, on numerous occasions, necessitated the help of community entities and social services. Many CM and TW SW had no money to pay rent, bills, or food. The pandemic plunged some of the participants into a situation of greater risk and, in some cases, forced them to devise new forms and places of work for survival. Despite their intention to respect safety measures, the use of a mask was impossible in intimate encounters, especially at the express request of clients. Likewise, during the lockdown, some CM and TW SW accepted risky practices for fear of not being able to support themselves. Even access to condoms was limited during the pandemic due to the temporary closure of NGOs. This increased the risk of acquiring STIs.

You realize that you have to expose yourself because it is the only way to survive. So, for example, we did not have an option other than making physical contact, and if you don’t, you starve. So, in my case, I resigned […]. I continued to attend to clients because I won’t say no, but I used a hydroalcoholic gel, wore a mask, and asked the client to shower first... Of course, there were no kisses, no blowjobs – none of that. Straight to [intercourse] and always using a condom. (P8. Trans woman)

Taking care of their health, both general and sexual, was part of a common practice that was already incorporated before the pandemic, such as the use of condoms, hygiene, medical check-ups, diet, and exercise. They also knew that the community entities were always available to help them manage the situation at all times. All the participants had also used their services at one time or another.

Financial problems? Yes, of course. Fighting to raise rent money was difficult, but it was achieved. It was difficult. It made you expose yourself a little more, didn’t it? (P4. Trans woman)

I liked the accompaniment [of the NGO], making friends, controlling for sanitation, and the treatment. The possibilities of giving us prophylactics to take care of ourselves, psychological help, and everything. Everything seemed fabulous to me. I have achieved what I needed to. (P8. Trans woman)

Given the difficulty of the situation, particularly in economic and social terms, the participants decided that the best option was to get used to the new situation, understand what was happening, and incorporate small, authorized interventions that allowed them to air their ideas and feel better, such as going for a walk, talking with friends on the phone, avoiding crowds, and healthy eating. In addition, some of the participants incorporated online sex work into their professional practice to survive in times of confinement and the pandemic safely.

I started looking for webcam options to work with; I started to see options for other things to say. I must get money from somewhere. If I don’t, I won’t survive. (P16. Trans woman)

I began posting videos on Only Fans® to see if something would come of it, and thus, provide a service. Even though it was very little money, at least it was income. It is something that does not fulfill me, and you have to be constant, but it is an option, and I use it. (P7. Cis man)

### Suggestions for Future Pandemic Situations: Increased Resources for Promoting Wellness Among Sex Workers

Participants requested a more comprehensive food dispensing network to cover more people. As mentioned, CM and TW SW were aware of the economic difficulties that NGOs have suffered for years. Consequently, they demanded more aid for these entities since it was they who provided most of the participants’ general and sexual health needs.

I think we need food, money, and a space to live in because I have a friend who gave her a room to be there, to pass the isolation period. Somewhere to rest, a safe place for sex workers to rest. (P24. Trans woman)

I have known you for four years, and I feel comfortable here. But we still need more help. The city council should give you more subsidies so that you can help us more. (P23. Trans woman)

The demands varied based on individual interests or basic needs ranging from increasing home care, expanding health service facilities, specific screening of COVID-19 for SW, distribution of financial aid to SW, to accommodation and other telemedicine options. Colleagues were also urged to fight for SW rights, particularly reinforcing activism to legalize sex work.

Laws still need to be promoted and actually implemented toward the transsexual community and those of us who work on this [SW]. Methods, laws, ways to help the transsexual community should be sought. Perhaps implementing laws for social and economic opportunities needs would help visibility. (P11. Trans woman)

## Discussion

Our findings highlight how the COVID-19 pandemic has impacted the bio-psycho-social health status of TW and CM SW in Barcelona, as well as their access to health and social services during the pandemic. The measures taken by the Government of Spain entailed: (a) discontinuity of some health services, including some HIV and STI care services; b) discontinuity in some associative and community services; c) Implementation, in some cases, of additional systems of care and follow-up of patients (e.g., telemedicine). The main difficulties in accessing and using health services in these populations during COVID-19 were structural, given the measures proposed by the Government (lockdown and social distancing). This is consistent with many international studies that reported that COVID-19 disruptions led to limited access to HIV/STIs and sexual and reproductive health services for SW in most countries (Gichuna et al., 2020; Howard, 2020; SWAN & ICRSE, 2020; UNAIDS, 2020a).

This study shows that structural barriers to health care access are frequent among CM and TW SW even before the pandemic. This finding is consistent with previous evidence in a different sociocultural context (Singer et al., 2020). Evidence shows that migrant sex workers experience important barriers in accessing preventive and curative interventions, mainly due to stigma, discrimination, and criminalization (Folch et al., 2008; Global Network of Sex Work Project, 2018). This study identifies that the main barriers to accessing health services for SW before COVID-19 are the CM and TW SW’s non-regular immigrant status and the waiting lists for health care access. It must be noted that most of the sex workers in Europe — most prominently in the West, South and North Regions of Europe, are migrants. In Catalonia, the proportion of migrants among a similar study among female sex workers 85%. Furthermore, the discriminatory experiences faced when accessing health services by some healthcare providers, and the difficulties in changing the registry name (in people with non-normative gender identities) increased the difficulties in accessing the system or doing it satisfactorily. Therefore, the COVID-19 pandemic has emphasized the existing structural violence experienced by TW and CM SW, mainly because of their multiple risks and the pandemic’s direct impact on their ability to work and provide for themselves. An intersectional approach (Crenshaw, 2021; Hill Collins & Bilge, 2016) to the phenomenon clearly identified several areas of oppression, such as gender identity, immigration status, economic status, ethnic background, and work. This lack of privileges has made TW and CM SW more exposed to violence, poorer health, and lack of government support (Sanders, 2016). In contrast, feelings of satisfaction with the care provided, the closeness and professional commitment of health professionals, the specialized services in STIs, and the support received from non-governmental organizations (NGOs), were identified as facilitating factors for health service access. Previous work has underscored the role of NGOs in helping people at risk to or living with HIV access and navigate the healthcare system in Spain (Gogishvili et al., 2021).

Despite the difficulties in accessing health services and professionals face-to-face, telematic visits partially covered this limitation. However, as described previously in the literature, the use of telehealth is not a 100% comprehensive solution to facilitate the provision of routine care. Moreover, the measures adopted, such as chronic medication delivery or health follow-ups, suffered some delays (Lieneck et al., 2021a, 2021b). On the other hand, NGOs had to respond flexibly to the needs of their service users and had to adapt existing services to a set of previously unanticipated restrictions. Consequently, SW could access and maintain general and sexual health services; therefore, there was general satisfaction with these services received during the pandemic. However, more personal help is necessary to cover economic deficiencies and aid so that NGOs can continuously respond to the needs of their users. This has also been previously reported in other countries (Pinto & Park, 2020).

Furthermore, it should not be overlooked that in addition to reduced access to services, this crisis negatively impacted the emotional health of SW. Their socialization patterns were modified, and they had to face labor and economic difficulties that generated psychological complications. As shown, many participants described feelings of loneliness and the need to be surrounded and cared for by their loved ones (mainly family and friends). In addition, a hard, uncertain, and difficult to manage future compelled emotional breakdowns (Esterwood & Saeed, 2020; Gossett & Hayward, 2020; Leyva-Moral et al., 2015). Therefore, it is necessary to provide for the psychosocial needs of this group.

During the COVID-19 pandemic, SW showed themselves to be self-responsible even though they were forced to get used to a new personal reality at work, were challenged financially, and had their physical and emotional health compromised. Thus, the pandemic became an exercise in the self-management of health, individual and collective responsibility, and solidarity. However, given the lack of privileges mentioned, some of them agreed to perform non-safe practices to gain some income. Therefore, our data indicate that multiple, co-occurring syndemic factors (mainly basic needs not being covered, mental health problems, and social isolation) placed these SW at a higher risk of COVID-19 and HIV/STIs, as reported previously among street-based SW (Rogers et al., 2021).

This study has a series of limitations that must be considered. First, the sampling used guaranteed the suitability of the profile of the participants in relation to the study’s objectives. However, it may have left some of the experiences of participants who did not show interest unexplored, either because of the difficulty of accessing the interviews or may be due to their discomfort with the topic discussed. Even so, the diversity of profiles and the redundancy of the data obtained guaranteed their credibility and dependability. Second, the collected data corresponded to the sociocultural reality of the given context, which does not allow for the generalization of the findings. However, the research team suspects several of the findings are generalizable to other sex workers populations internationally. Third, the interviews were conducted by three different professionals, which could contribute to the variability during data collection. Various consensus and training meetings were held to achieve homogenization of the data collection methods; however, the methodology used allows for certain adaptations of the interview script based on the communicative characteristics of the interviewees. Finally, there might be some information bias due to the sensitivity of the topic, which was minimized, to generate a safe environment of confidentiality and non-judgment by the interviewers.

## Conclusions

The main difficulties regarding the access and use of health services in TW and CM SW during COVID-19 were structural, given the measures adopted by the Government. Telematic visits partially covered the limitations of accessing health services and professionals. However, there were delays in services, specifically in the delivery of chronic medication and poor control of some health episodes. The main barriers to health service access for SW before COVID-19 were the illegal immigration situation and the waiting lists for health care services. Furthermore, the discriminatory experiences of professionals and the difficulties in changing the registry name (in people with gender diverse identities) increased the difficulties in accessing the system or doing it satisfactorily. On the contrary, the closeness and professional commitment of health professionals and specialized services in STIs and the support received from NGOs were identified as facilitating factors to health service access.

The COVID-19 pandemic has made it difficult to access health and social services, negatively impacting health experiences. SW were dissatisfied with the measures implemented during the pandemic by the Government (specially lockdown, changes in health centers dynamics, and no consider their needs during decision-making); they believe those measures were the main responsible of their labor and economic problems. Consequently, this has also increased their risk of exposure to the virus. The adaptation of health services, such as telemedicine, although considered better than the cancelation of visits, was not sufficient to meet their health needs. Due to the interventions of NGOs, CM and TW SW were able to maintain their access to general and sexual health services during the pandemic and were satisfied with these services. However, they call for more personal assistance and aid for NGOs to continue providing health services and support. Moreover, it should not be overlooked that this crisis had negatively impacted the emotional health of SW since their socialization patterns were modified, and they had to face labor and economic difficulties. Therefore, it is necessary to provide for the psychosocial needs of this group.

These results highlight the importance of considering CM and TW SW as a target population for public health policymakers and health practitioners, given the risks identified in this study and previous evidence. Specific research on the risks and vulnerabilities by type of work will be needed (street, online), due to the different practices and needs of those who operate in the street and/or online may face. Moreover, health promotion with a person-centered and intersectional approach is suggested for future research and practice. This will help provide easier access to health and social services and promote non-discriminating/stigmatizing environments. The work carried out by NGOs during the pandemic is an example of this type of approach that responded to the difficulties of accessing the public health system accentuated by the pandemic. Furthermore, determinants of health—economic income, nutrition, and affordable housing—must be considered in future research and practice; thus, dyad healthcare and economics must be studied together.

## Data Availability

Due to the nature of this research, participants of this study did not agree for their data to be shared publicly, so supporting data are not available.
